# TGF-β Enhances the Anti-inflammatory Effect of Tumor-Infiltrating CD33+11b+HLA-DR Myeloid-Derived Suppressor Cells in Gastric Cancer: A Possible Relation to MicroRNA-494

**DOI:** 10.31557/APJCP.2020.21.11.3393

**Published:** 2020-11

**Authors:** Mai Moaaz, Hassan Lotfy, Bassem Elsherbini, Mohamed A. Motawea, Geylan Fadali

**Affiliations:** 1 *Department of Immunology and Allergy, Medical Research Institute, Alexandria University, Alexandria, Egypt. *; 2 *Department of Surgery, Vascular Surgery Unit, Faculty of Medicine, Alexandria University, Alexandria, Egypt. *; 3 *Department of Experimental Surgery, Medical Research Institute, Alexandria University, Alexandria, Egypt. *; 4 *Department of Pathology, Medical Research Institute, Alexandria University, Alexandria, Egypt. *

**Keywords:** Myeloid-derived suppressor cells, gastric cancer, transforming growth factor beta, microRNA-494

## Abstract

**Background:**

Accumulation of myeloid-derived suppressor cells (MDSCs) constitutes a key mechanism of tumor immune evasion in gastric cancer (GC). Therefore, searching for more accurate prognostic factors affecting their immunosuppressive role has become a growing interest in cancer immunotherapy research. Increased expression of microRNA-494 was noticed in MDSCs from tumor-bearing mice, suggesting another new therapeutic objective for cancer treatment. It was also discovered that tumor-derived transforming growth factor beta (TGF-β) is responsible for the up-regulation of microRNA-494 in MDSCs. The purpose of this study was to address the effect of recombinant (rTGF-β) on the anti-inflammatory activity of MDSCs in GC and its possible association with micro-RNA-494 expression in tumor tissue.

**Methods:**

Freshly obtained GC tumor tissue samples and peripheral blood were used for isolation of CD33+11b+HLADR- MDSCs cells from 40 GC patients and 31 corresponding controls using flow cytometry. MDSCs were co-cultured with isolated autologous T cells to assess proliferation and cytokine production in the presence and absence of rTGF-β. Real-time PCR and Enzyme linked immunosorbent assay were used to evaluate tumor expression of miRNA-494 and TGF-β respectively.

**Results:**

Results showed that rTGF-β markedly increased the suppressive ability of tumor MDSCs on proliferation of autologous T cells and interferon gamma production. However, no inhibitory effect was observed for MDSCs from circulation. In addition, infiltration of MDSCs in tumors is associated with the prognosis of GC. MiRNA-494 was also extensively expressed in tumor samples with a significant correlation to MDSCs.

**Conclusion:**

These results indicate that tumor-derived MDSCs but not circulatory MDSCs have an immunosuppressive effect on T cells, potentially involving TGF-β mediated stimulation. Results also suggest a role for miRNA-494 in GC progression. Therefore, control of TGF-β and miRNA-494 may be used as a treatment strategy to downregulate the immunosuppressive effect of MDSCs.

## Introduction

Gastric cancer (GC) is considered as the fourth most common cancer in men and the fifth in women worldwide. Almost one million new cases are diagnosed every year; 70% of them occur in developing countries (Sitarz et al., 2018). 

One of the most significant findings that originated from hundreds of unsuccessful attempts at immunotherapy was the highly immunosuppressive nature of the tumor microenvironment (ME), both at the tumor site and in the circulation. The infiltration of various immune cells in tumors has been shown to be associated with the prognosis of GC (Labani-Motlagh et al., 2020). Among them are the immunosuppressive myeloid cells that promote tumorigenesis (Law et al., 2020). 

Myeloid-derived suppressor cells (MDSCs) are a heterogeneous population of cells comprising cells of the myeloid lineage at several stages of differentiation. They accumulate in blood, lymph nodes, bone marrow, and tumor sites in cancer patients, and are capable of inhibiting both innate and adaptive immune responses (Bergenfelz et al., 2020). Human MDSCs express common myeloid markers; CD11b and CD33 but lack HLA-DR (Bronte et al., 2016). Indeed, one of the central mechanisms of tumor evasion to the immune response is the accumulation of MDSCs. Their increased circulating numbers and their infiltration in tumor tissue influence the progression of tumors via different mechanisms including: local immune suppression and stimulation of tumor neovasculogenesis (De Cicco et al., 2020). 

Substantial research concluded that, MDSCs are recruited and activated by tumor-derived cytokines and growth factors, such as interleukin (IL)-2, IL-6, IL-1b, stem cell factor (SCF), granulocyte monocyte colony stimulating factor (GM-CSF), G-CSF, and vascular endothelial growth factor (VEGF) (Lim et al., 2020). In addition, tumor production of transforming growth factor beta (TGF-β) may affect MDSCs differentiation (Lee et al., 2018), leading to the increment of their frequency (Santibanez and Bjelica, 2017). 

Bodogai et al., (2015) suggested that TGF-β contributes to MDSC immunosuppressive properties via the activation of B regulatory cells (Breg), which can educate MDSC to suppress T cell proliferation. Another suggested mechanism in TGF-β-induced MDSC is its capability to regulate miRs expression (Santibanez and Bjelica, 2017). MicroRNAs (miRNAs) have emerged as important regulators of myeloid lineage development and differentiation (El Gazzar et al., 2012). They are small non-coding RNAs that post-transcriptionally modulate the expression of multiple target genes and are hence implicated in a wide series of cellular and developmental processes (He and Hannon, 2004). Recent studies have begun to unravel the emerging role of individual miRNAs in MDSC functional accumulation during tumor development as they negatively mediate the differentiation and activity of tumor MDSCs (Su et al., 2019).

One specific miRNA, miRNA-494, is a major modulator of the cell cycle progression from gap 2 (G2) to mitosis (M) (Liu et al., 2012). Increased expression of miRNA-494 was noticed in MDSCs from tumor-bearing mice, suggesting another possible new therapeutic objective for cancer immunotherapy. In addition, the manipulation of miRNA-494 influenced migration of MDSCs and their apoptosis (Chen et al., 2015). It was also reported that TGF-β derived from tumors is responsible for miRNA-494 up-regulation in MDSCs (Liu et al., 2012). 

Alternatively, MDSCs have the ability to secrete TGF-β that has direct immunosuppressive effects within the tumor ME and induces regulatory T cells (Treg), which suppresses tumor-specific T cell responses (Batlle et al., 2019; Salminen et al., 2019). Elevated TGF-β in serum and tumor tissue was correlated with prognosis in gastrointestinal cancers (Katz et al., 2016). Besides, recent studies have revealed dual roles in gastrointestinal cancer initiation and progression, as it functions as a tumor suppressor during early-stage carcinogenesis and as a tumor promoter during later stages. Therefore, the development of TGF-β–based therapeutics is challenging; however, the future of TGF-β pathway-based strategies against GC is promising (Luo et al., 2019). 

Although MDSCs cells have been characterized in various types of cancers, the significance of these cells in GC remains to be elucidated. Herein, we investigated the interactions of these cells in mediating clinical parameters of GC as well as the inhibition of T cell proliferation and interferon gamma (IFN-γ) production under the effect of recombinant TGF-β. In addition, we explored the relation between these cells and TGF-β and miRNA-494 in GC.

## Materials and Methods

*Ethics*

The study conforms to the Declaration of Helsinki for use of human tissue or subjects and the study protocols as well as the collection and use of blood samples and tissues were previously approved by the Medical Ethical Committee of Alexandria University and bioethical research committee approval was taken (ethics board approval number: IRB NO.: 00007555-FWA NO: 00015947). A written informed consent was taken from all patients and healthy donors prior to blood sampling and/or tumor tissue harvesting. 

*Subjects*

Blood samples were collected from Age- and sex-matched healthy volunteers (N = 31) and patients with GC (N = 40) who were seen at the Department of Surgery in Alexandria University Hospital and Medical Research Institute, Alexandria University, collected before any treatment was initiated. All patients were diagnosed with GC for the first time with histological confirmation of the diagnosis. None of these patients had received therapy prior to blood donation, and all had progressed at the time of immune testing. Exclusion criteria included a history of recent steroid treatment, autoimmune disease, or history of chronic hepatitis B or C. No control subject had a previous history of cancer. 

*Cell isolation from peripheral blood*

Peripheral venous blood samples were drawn from healthy donors and cancer patients into heparin coated vacutainers for separation of peripheral blood mononuclear cells (PBMCs) (Fuss et al., 2009). The diluted sample was over layered gently over half its volume of Ficoll-hypaque (1077) (Sigma-Aldrich) and centrifuged at 1,800 rpm for 30 minutes at room temperature. After centrifugation, the band of PBMCs was aspirated; washed twice with PBS containing 1% of fetal calf serum, pelleted and resuspended in 1 ml RPMI 1640 (Biochrom KG Berline), to determine the cell count and viability using a hematocytometer and Trypan blue dye exclusion.

*Tissue Samples*

Freshly resected tumors, matched paraneoplastic tissues and lymph nodes (LN) were obtained immediately after surgical resection from patients with GC. Each sample tissue was divided into two parts: one part for the routine histopathological studies, and Haematoxylin and eosin (H&E) staining. The other part was maintained in organ transportation medium on ice until used for the tissue slicing and culture.

*Cell isolation from tumor site and lymph nodes*

For isolation of immune cells infiltrating tumor, surrounding paraneoplastic tissue and LN tissues, 100 mg of tissues were cut into pieces then were enzymatically digested in RPMI 1640 medium containing 500 mg/ml liberase and 200 mg/ml DNase (Roche) for 45 min at 37 C with gentle shake every 10 min until all the tumor tissue had resolved into a cell suspension. After that, the resulting cell suspension was filtered through 70 mL cell strainers (BD Biosciences) and subjected to the density centrifugation over Ficoll Hypaque as described above. 

*Identification of Myeloid derived suppressor cells by flow cytometry*

For identification of circulating and tissue infiltrating CD33+11b+HLA- MDSCs, (Freeman et al., 1995), cells were tested on a BD FACS Calibur flow cytometer (FACSCalibur, Becton-dickinson, USA). Direct fluorescent staining was done on surface molecule HLA-DR using anti-HLA-DR phycoerythrin (PE; clone GRB1, mouse IgG2a, Immunostep, Spain), then CD33, CD11b in double settings using anti-CD33 phycoerythrin (PE; clone HIM3-4, mouse IgG1, Immunostep, Spain) and anti-CD11b fluorescein isothiocyanate (FITC; clone DCIS1/18, mouse IgG2a, Immunostep, Spain) on CD3+CD4+ cells (anti-CD3 phycoerythrin (PE; clone HIM3-4, mouse IgG1, Immunostep, Spain), anti-CD4 (FITC) (BD biosciences) to determine CD33+ and CD11b+ cells within HLA-DR- population. It was analyzed using Cell Quest software (Becton-Dickinson). Gated cells subpopulations that were analyzed were approximately 10^4^ cells after exclusion of the autofluorescence. 

*In vitro suppression assay*

T lymphocyte and MDSCs isolation

For co-culture experiments, autologous T cells were immediately isolated from PBMCs using a Pan T cell isolation kit II for further separation of CD3+ T-cells (CD3-labeled magnetic beads; MACS, Milteny Biotec, Bergisch Gladbach, Germany) as described elsewhere. The purity of the cells after sorting was 95%. For functional analysis, purified CD3+T cells were labeled with 2 µM of Carboxyfluorescein Succinimidyl Ester (CFSE) (Invitrogen; Thermo Fisher Scientific, Inc.) according to the manufacturer’s instructions.

CD33CD11b+HLA- MDSCs were isolated from both PBMCs and tumor tissues of each patient using MACS magnetic bead isolation kits (Miltenyi Biotec), according to the manufacturer’s instruction. 

*Co-culture of MDSCs and T lymphocyte*

Freshly sorted MDSCs were co-cultured with autologous CD3+ T cells for 3 days in 5% CO_2_ incubator at 37°C. Briefly, T cells (104 /well) were incubated in 100 µl of the supplemented RPMI- 1640 tissue culture medium and stimulated with 10 µl (10 µg/ml) phytohaemagglutinin (PHA), anti-CD3 (2 mg/mL) (eBioscience, Cat. No. 14-0037-82) and anti-CD28 (0.5 mg/mL) (all from eBioscience). Four sets of three wells each were then considered in 96- well plates. No MDSCs were added to the first set, 50,000 autologous MDSCs isolated from PBMCs were added to the second set of stimulated T cells, 50,000 autologous MDSCs isolated from tumor tissue were added to the third set, and 50,000 MDSCs + recombinant TGF-β (rTGF--β ) (T-7039; Sigma-Aldrich Product Number: MFCD00166089) were added to the fourth set at the concentrations indicated. 

*Suppression assay*

Suppressor effect on responder T cells was tested by two different methods, as follows: CFSE dilution and MTT (Cat. No.: 11465007001 Roche Diagnostics GmbH; Germany). After 3 days, cells were harvested, assessed for proliferation with CFSE dilution as described previously Percentage suppression was calculated by dividing the number of proliferating CFSE-diluting responder cells in the presence of different ratios of responder: suppressor cells by the number of proliferating responder cells when cultured alone, and multiplied by 100. 

The MTT was weighed and then dissolved in 50 μl of Dimethyl Sulfoxide (DMSO). Then, to make a final solution of 10 mg/ml, RPMI medium was added. MTT reagent (10ml) was then added to each well; so 100 μl was present per well. It was then incubated for 4 h at 37 °C in the presence of 5% CO_2_ in the incubator. After 4 h, the plates were gently tilted and the supernatant was removed, and placed in a microcentrifuge tube. 100 μ l of DMSO was added to the remaining sample in the well. The tubes were subjected to centrifugation at 2,000 rpm for 10 min. After centrifugation, the supernatant was discarded and 100 μl of solution DMSO was added to the centrifuge tube and transferred to the well. An enzyme-linked immunosorbent assay (ELISA) reader was then used for reading each well at OD 570 nm. Cells were harvested, assessed for proliferation with MTT (3-(4,5-dimethylthiazol--yl) -2,5-diphenyltetrazolium bromide).

*Interferon gamma (IFN-γ) production*

Level of IFN-γ production was determined by ELISA in the supernatants. This was done using an IFN-γ OptEIA kit (BD Bioscience).

*RNA extraction and real-time RT-PCR*

Total RNA including small RNAs was extracted from cultured tumor/surrounding cells and blood samples of controls using miRNeasy FFPE mini Kit and QIAzol lysis reagent (Qiagen cat no. 217004) according to the manufacturer’s instructions. The concentration of RNA was determined using a spectrophotometer and the RNA integrity was verified using Agilent 2100 BioAnalyzer (Agilent Tech, Palo Alto, CA). The levels of miRNAs-494 were measured by real-time quantitative RT-PCR (qRT-PCR) using TaqMan^®^MicroRNA Assays (applied biosystems). For mRNA detection, reverse transcription was performed with TaqMan^®^ MicroRNA Reverse Transcription Kit (Applied Biosystems PN 4366596) and according to the manufacturer’s instructions. The real-time PCR reaction contained: 10 μL of TaqMan^®^ 2 μL Universal PCR Master Mix, No AmpErase^®^ UNGb (Applied Biosystems PN 4324018) , 1μL of each of sense and anti-sense primers, in addition to 2 μL of cDNA template and 6 μL of H_2_O. The primers for miRNA-494 were as follows: 5’-GAGGTTTCCCGTGTATGTTTCA-3’.The program of two step real time RT-PCR was 95°C for 10 minutes, followed by 40 cycles of 95°C for 15 seconds, and 60°C for 60 seconds. The relative expression level of mRNA-494 was normalized to that of internal control β-actin by using the 2^-ΔΔCt^ cycle threshold method. 

*Tumor TGF-β production*

 Cytokine production was determined in the supernatant by ELISA using TGF- β kit (BD Bioscience) according to manufacturer’s instructions.

*Statistical analysis*

All data were presented as mean and SD (standard deviation of mean) they were compared with the tabulated probability value (P value) as the 0.05 level using IBM SPSS software package version 20.0. (Armonk, NY: IBM Corp). Level of P value was considered as significant if it is below 0.05. The Kolmogorov-Smirnov test was used to verify the normality of distribution Quantitative data. The following statistical tests were used: Chi square test, Mann-Whitney, F test (ANOVA), Post Hoc Test (adjusted Bonferroni), Student t-test, Paired t-test, Pearson coefficient test and linear correlation coefficient “r” to examine the relationship between different parameters. 

## Results

*Subjects’ characteristics*

We analyzed PBMCs and tumor tissues of 40 patients with gastric cancer and 31 age-/ sex-matched controls (23 males and 8 females). Patients characteristics are shown in Table 1 as well as the comparison between the studied groups according to demographic and laboratory data.

*Circulatory MDSCs expression and correlation to clinico-pathological parameters*

**Figure 1:**
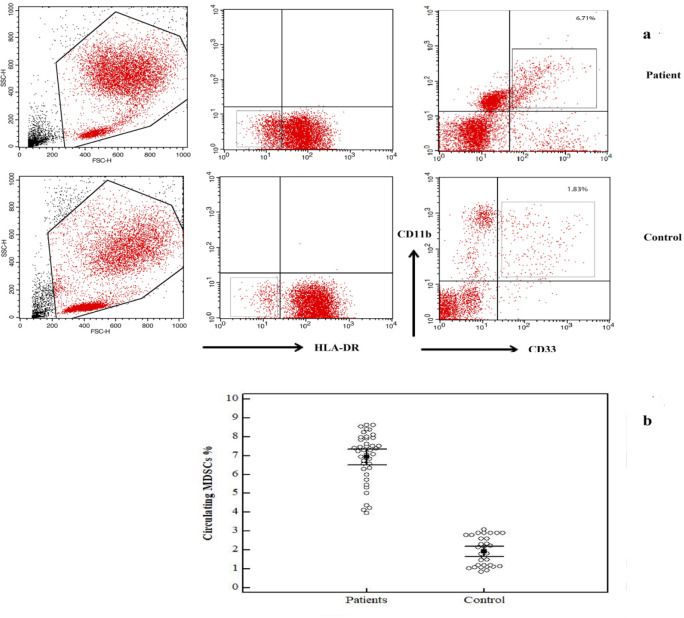
Comparison between GC Patients and Control Group in CD11b+CD33+HLA- MDSCs (%): Percentages of CD33+11b+HLA-DR- MDSCs cells from GC patients and controls: a: Isolated cells from PBMCs from a GC patient were stained with anti-HLA-DR, anti-CD33, and anti-CD11b. b: MDSCs cells in healthy control subject. C: Data represented as means ± SDs of percentages of CD11b+CD33+HLA- cells using flow cytometry, where isolated cells from patients (left) and controls (right) were stained with anti-HLA-DR, followed by anti-CD33 and anti-CD11b. Data were summarized as dot plot. Each dot represents the expression percentage of MDSCs for one individual. The level of significance was set at *p ≤0.05

Analyzed by flow cytometry, CD11b+CD33+HLA- MDSCs percentages in peripheral blood were found to be significantly elevated in gastric cancer patients (6.93 ± 1.30 %) than control subjects (1.92 ± 0.77 %, p<0.001) (Figure 1).

Due to the heterogeneity of gastric cancer severity and clinical variation, and in order to elucidate the relationship between MDSCs and susceptibility to cancer progression and prognosis, we conducted further statistical analyses. We analyzed the interactions between percentages of MDSCs in peripheral blood and known tumor criteria: cancer stage and grade as well as metastasis to lymph nodes and vascular invasion. 

*Cancer stage*

**Figure 2:**
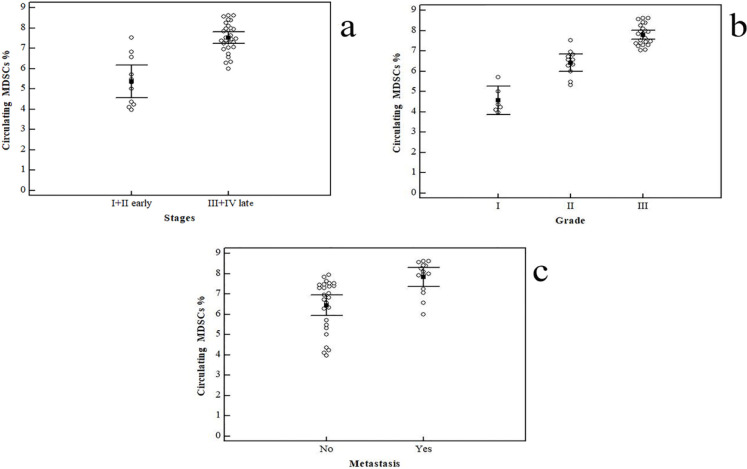
Relation of Circulating CD11b+CD33+HLA- MDSCs to GC Characteristics. a: Comparison between (stage I + II) and (stage III + IV) in percentages of circulating CD11b+CD33+HLA- cells: Data represented as means ± SDs and were summarized as dot plot. Each dot represents the percentage of MDSCs for one individual. The level of significance was set at *p ≤0.05. b: Comparison between Grade I, II, and grade III in percentages of circulating CD11b+CD33+HLA- cells. c: Comparison between (patients with no metastasis) and (patients with metastatic disease) in percentages of circulating MDSCs

Figure 2a shows a correlation between cancer stage and the percentage of MDSCs in PBMCs: higher levels of MDSCs were found in patients with stage III (7.23 ± 0.49 %; n = 15) or IV (7.83 ± 0.81%; n = 14) compared with those with an early stage of disease (stage I =4.39 ± 0.44%; n = 4; or stage II = 5.93 ± 1.13 %; n= 7) with high statistically significant difference, p<0.001.

*Tumor grade*

This increase was correlated with tumor grade: GC patients with grade III (n= 23) had higher levels of circulating MDSCs compared with those with grade I (n= 6) or II (n= 11) cancer [7.79 ± 0.50 % versus (4.57 ± 0.67 % and 6.41 ± 0.64 % respectively), p<0.001] (Figure 2b).

*Metastasis*

In 26 patients with no metastasis, the percentage of MDSCs in the PBMCs was significantly lower compared with those with metastatic disease (n=14) (6.45 ± 1.26 %versus 7.83 ± 0.81%, p <0.001) (Figure 2c). 

*Lymph node involvement*

It was noteworthy that patients with lymph node involvement showed increased percentages of circulatory MDSCs; those with N2 had (7.17 ± 0.91%; n= 23), N1 (5.84 ± 1.50; n= 7), and N3 with (7.88 ± 0.52%; n= 8). Two patients with no metastasis to the lymph nodes (N0) showed the least MDSCs expression (4.17 ± 0.28%). 

*Tissue infiltrating MDSCs and their correlation to clinico-pathological parameters*

**Figure 3:**
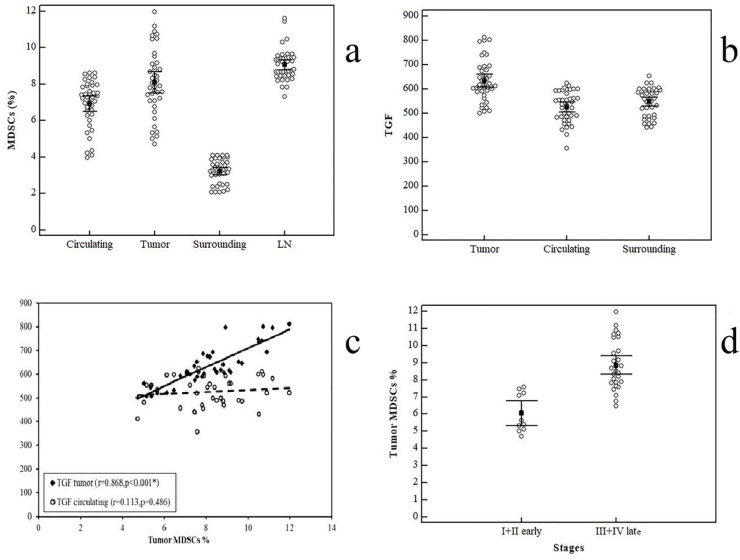
a: Comparison between CD11b+CD33+HLA- cells in different types of samples. b: Comparison between TGF-β levels (pg/ml). c: Correlation analysis between CD11b+CD33+HLA- cells in tumor tissues and TGF-β level in both circulation and tumor tissues. Correlation was conducted by Spearman coefficient. d: Comparison between (stage I + II) and (stage III + IV) in percentages of tumor derived CD11b+CD33+HLA- cells: Data represented as means ± SDs and were summarized as dot plot. Each dot represents the percentage of MDSCs for one individual. The level of significance was set at *p ≤0.05

MDSCs showed a significantly increased expression in tumor specimens (8.09 ± 1.83%; n=40) than paraneoplastic surrounding tissue (mean ± SD = 3.22 ± 0.64%) with p<0.001, and circulatory MDSCs (Fig.3a). This increased expression is obviously accompanied with elevation of TGF-β production in tumor tissues (633.6 ± 84.47 pg/ml) than surrounding apparently normal tissues (548.2 ± 58.09, p <0.001) (Figure 3b). In line with these observations, our analysis of tumor- infiltrating MDCSs confirmed the direct association of these cells with TGF-β (Figure 3c). These results suggest that TGF-β may be associated with production of MDSCs in GC patients.

Interestingly, lymph nodes specimens from patients with gastric cancer showed a statistically significant elevation in MDSCs expression than tumor tissue MDSCs (9.06 ± 0.87%; n=38, p<0.001).

Patients with metastatic disease showed a significant interaction with an increased MDSCs percentages (9.83 ± 1.39%) than those with non- metastatic disease (7.16 ± 1.26, p=0.003). Also, patients with late stages showed higher percentages of tumor- infiltrating MDSCs than those with early stages (Figure 3d). 

*TGF-β level does not correlate to clinico-pathological parameters*

**Figure 4:**
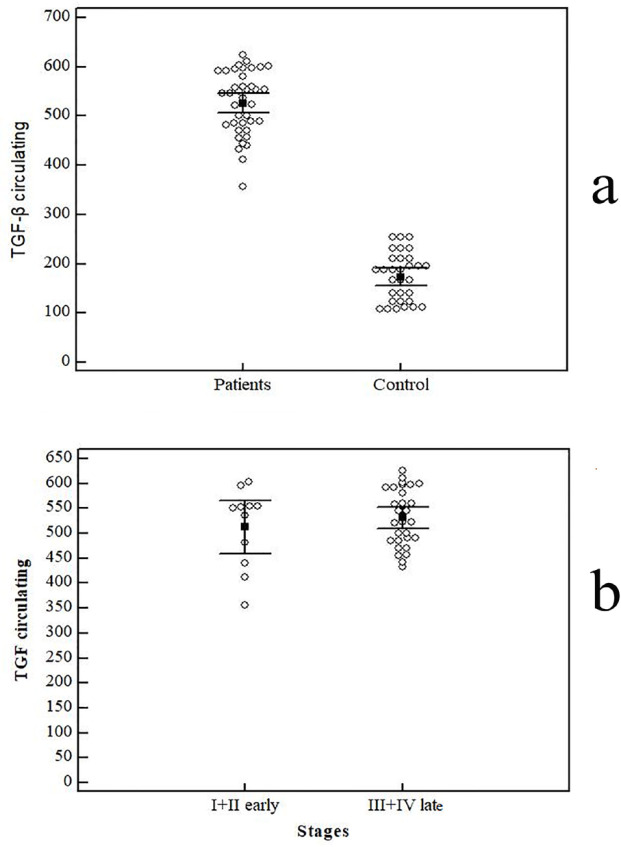
a: Comparison between GC patients and control group in TGF-β levels in circulation (pg/ml). b: Comparison between (stage I + II) and (stage III + IV) in TGF-β levels in circulation (pg/ml). Data represented as means ± SDs and were summarized as dot plot. Each dot represents the level for one individual. The level of significance was set at *p ≤0.05

We further assessed TGF-β cytokine concentrations as shown in Figure 4a. Our results showed that patients had an elevated level of TGF-β concentration (526.28 ± 63.63 pg/ml) than controls (172.90 ± 49.98 p`g/ml).

Of note, we did not notice any positive correlation between the level of TGF-β and cancer stage. Stage I (n= 4) had mean of TGF-β (495.25 ± 62.91 pg/ml), Stage II (522.57 ± 90.67; n= 7), Stage III with (525.13 ± 59.85 pg/ml; n= 15), and finally patients represented with stage IV (538.21 ± 55.86 pg/ml; n= 14; P=0.704) (Figure 4b).

Moreover, there was no significant interaction between metastatic involvement and the cytokine level [(non-metastatic = 519.85 ± 67.60 pg/ml) vs (metastatic = 538.21 ± 55.86 pg/ml); p=0.391)]. Neither of tumor grade (p=0.501) nor the LN involvement (p=0.499) showed any significant relation to TGF-β levels.

*Role of miRNA-494 expression and its correlation to MDSCs and clinico-pathological parameters*

**Figure 5:**
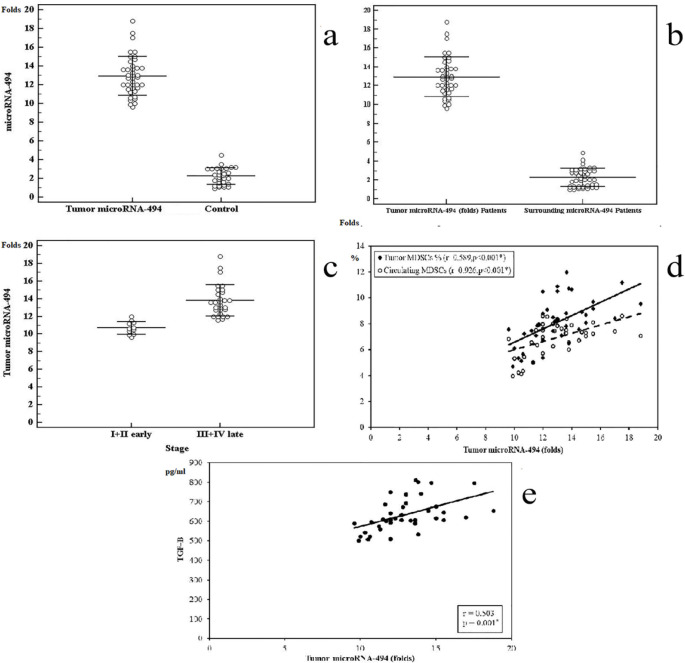
. a: Comparison between GC patients and control group in microRNA-494 expression (folds). b: microRNA-494 expression (folds) in tumor tissues and their surrounding tissue (n=40). c: Comparison between (stage I + II) and (stage III + IV) in microRNA-494 expression (folds). Data represented as means ± SDs and were summarized as dot plot. Each dot represents the expression for one individual. d: Correlation analysis between CD11b+CD33+HLA- cells in tumor tissues and circulation vs microRNA-494 expression (folds). e: microRNA-494 expression (folds) vs TGF-β level. Correlation was conducted by spearman coefficient. The level of significance was set at *p ≤0.05

We focused our subsequent efforts in identifying the role of miRNA-494 in GC. Using qRT-PCR, we found a significant increase in the expression of miRNA-494 in GC patients compared with healthy controls [(12.94 ± 2.10 folds) versus (2.25 ± 0.89 folds), p=0.001)] (Figure 5a). Additionally, miRNA-494 was extensively expressed in tumor samples (range = 10-18 folds; mean ± SD = 12.94 ± 2.10) than surrounding paraneoplastic tissue (range = 1-4.5 folds; mean ± SD =2.27 ± 0.97, p=0.01) (Figure 5b). This increase correlated with cancer stage: GC patients with late stages (stage III or IV cancer) had higher levels of miRNA-494 compared with those with early stages (stage I or II cancer) [(13.79 ± 1.78; n = 27) versus (10.69 ± 0.74; n = 13) folds] (Figure 5c). Interestingly, we also found a positive correlation between the numbers of tumor derived and circulating MDSCs and miRNA-494 (Figure 5d). This result suggests that miRNA-494 may be associated with production of MDSCs in GC patients. Furthermore, TGF-β was correlated directly to miRNA-494 (Figure 5e).

*In vitro immunosuppressive effect of tumor-derived MDSCs*

**Figure 6:**
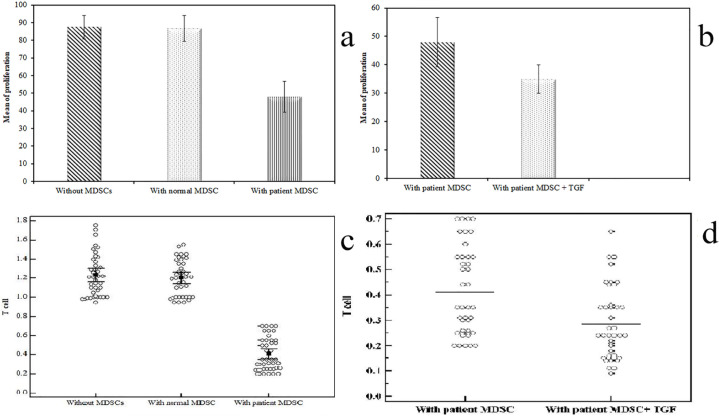
a: CD11b+CD33+HLA- cells were mixed with CFSE-labeled T cells (responder T cells), and the proliferation of responder T cells was assessed by the percentage of CFSE diluting responder T cells. n = 40. b: rTGF-β effect on the suppressive activity of tumor derived CD11b+CD33+HLA- cells: c and d: T cell proliferation was assessed by MTT. Data represented as means ± SDs and were summarized as dot plot. Each dot represents the level for one individual. The level of significance was set at *p ≤0.05

**Figure 7:**
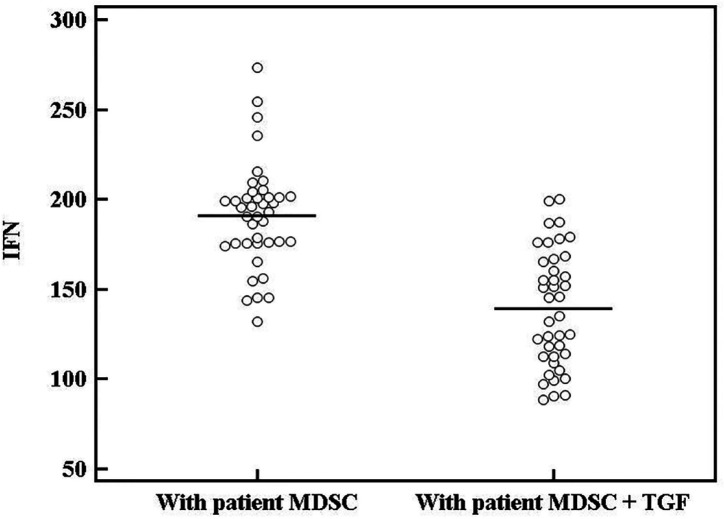
rTGF--β effect on the suppressive activity of MDSCs on IFN-γ release from responder T cells. Data represented as means ± SDs and were summarized as dot plot. Each dot represents the serum level of IFN-γ for one individual. Left dot plot shows unstimulated IFN-γ (pg/ml). Right dot plot shows cytokine level after addition of rTGF-β. The level of significance was set at *p ≤0.05

To determine if MDSCs inhibit the activity of T cells, we purified MDSCs from tumor tissues of cancer patients and from their PBMCs, and then we co-cultured them separately with autologous T cells at the 1:2 ratios in the presence of CD3/CD28 antibody stimulation for 72 hours. We further assessed proliferation of T cells using the extent of CFSE dilution of labeled autologous T cells (hereafter called responder cells). As illustrated in Figure 6a, the results of CFSE staining demonstrated that purified MDSCs from tumor samples markedly suppressed the division of autologous T cells and are able to suppress the proliferative response of activated responder cells. The suppressive capacity of MDSCs cells was also assessed by measuring their capacity to suppress the proliferation of responder cells determined by MTT. Results from one representative experiment are depicted in Fig.6b. Simultaneously; IFN-γ production was also inhibited as assessed by ELISA (Figure 7). However, no inhibitory effect was observed for circulating MDSCs in T cells and IFN-γ production. The result indicates that tumor-derived MDSCs but not MDSCs from PBMCs have the immunosuppressive effect on T cells. 

*The role of rTGF-β in enhancing MDSCs immunosuppressive ability in vitro*

MDSCs in GC showed enhanced suppression of IFN-γ production when we added recombinant TGF-β to the co-culture (177.0 ± 25.33 pg/ml; p =0.009) with a further suppression of T cells proliferation [By MTT: 0.28 ± 0.14; by CFSC: (34.90 ± 5.01%)] (Figure 6). This enhanced suppression was obviously seen in late cancer stages (p = 0.034); however, it showed no relation to cancer grade (p =0.154). 

**Table 1:** Subjects’ Characteristics

Characteristic	Patients	Control	p
	(n = 40)	(n = 31)	
Age	59.63 ± 8.98	58.45 ± 7.35	0.557
≥60	21 (52.5%)	11 (35.5%)	0.153
<60	19 (47.5%)	20 (64.5%)	
Sex			
Male	30 (75.0%)	23 (74.2%)	0.938
Female	10 (25.0%)	8 (25.8%)	
Tumor grade			
1	6 (15.0)		
2	11 (27.5)		
3	23 (57.5)		
Cancer Stage			
I	4 (10.0%)	–	–
II	7 (17.5%)	–	
III	15 (37.5%)	–	
VI	14 (35.0%)	–	
Metastasis			
Negative	26 (65.0%)	–	–
Positive	14 (35.0%)	–	
L.N			
N0	2 (5.0%)	–	–
N1	7 (17.5%)	–	
N2	23 (57.5%)	–	
N3	8 (20.0%)	–	
Duration of illness (months)	5.40 ± 2.80	–	–
Hemoglobin (g/dl)	9.91 ± 1.47	12.78 ± 1.05	<0.001*
White Blood cell count	7.71 ± 2.20	10.28 ± 3.33	<0.001*
Differential white cell count			
Basophils (%)	0.19 ± 0.20	0.32 ± 0.09	0.001*
Neutrophils (%)	64.33 ± 12.36	67.28 ± 2.34	0.365
Lymphocytes (%)	21.30 ± 5.86	23.62 ± 1.06	0.002*
Eosinophil’s (%)	3.76 ± 2.18	0.52 ± 0.03	<0.001*
Liver Function			
SGOT (ALT) u/l	20.37 ± 9.07	21.35 ± 1.14	<0.001*
SGPT (AST) u/l	23.42 ± 11.39	32.94 ± 1.65	0.501

p, p value for comparing between Patients and Control; *, Statistically significant at p ≤ 0.05

## Discussion

One of the crucial aims in novel immunotherapeutic strategies is to elevate the levels of anti-tumor infiltrating T lymphocytes and their protective cytokines (Vignali and Kallikourdis, 2017). MDSC is considered a key checkpoint, a T cell suppressor, and a critical player in mediating cancer immune evasion (Zhou et al., 2016). Moreover, studies have shown an expansion of infiltrating myeloid cells in different types of cancers (Rodriguez et al., 2009; Liu et al., 2010; Arihara et al., 2013; Toor et al., 2016; Toor et al., 2017).

In the present study, we compared MDSCs in the peripheral blood of 40 patients with GC versus 31 controls. In line with the previous results, we also reported an expansion of circulating myeloid cells in GC patients versus age and sex-matched controls. Moreover, in the present analyses, we have established further that GC patients have higher levels of myeloid cells in tumor tissue than circulation. To the best of our knowledge, only three published studies have explored the clinical significance of intratumoral MDSCs (Diaz-Montero et al., 2009; Cui et al., 2013; Najjar et al., 2017). 

Interestingly, we found that levels of MDSCs in LN are higher than in both tumor and normal stomach tissue. Our observation included that MDSCs are present in the premalignant surroundings as well as the malignant tumor microenvironment (Ma et al., 2019). 

In this study, we sought to assess whether tumor MDSCs frequencies correlate with cancer parameters, and we reported that expansion of circulating MDSCs in GC patients correlated with advanced stage and histological grade, which suggested their role in tumor progression (Toor et al., 2016). However, no such correlation was observed in breast cancer studies (Toor et al., 2017). Concordant with previous reports (Zhang et al., 2013; Ohki et al., 2012), we found that GC patients with poorly differentiated tumors have significantly higher levels of MDSCs than those with well-defined tumors. Therefore, our data suggest that MDSCs levels in GC are dependent on patient’s clinical presentation as reflected by the tumor grade, and disease stage. 

When dividing patients into patients with metastasis or without metastasis, we found that patients with metastasis had the significantly elevated percentage of MDSCs and they also correlated with presence L.N involvement. Our results are consistent with those seen in experimental animal cancer models and human cancer showing levels of MDSCs correlate linearly with tumor burden (Melani et al., 2003; Walter et al., 2012; Diaz-Montero et al., 2014). While several studies have shown a correlation between peripheral MDSC levels and patient outcomes, to our knowledge, ours is the first to show a correlation between MDSC subset levels and metastatic disease and LN involvement in GC. 

In many cancer patients, the lack of efficacy of several immunotherapeutic approaches, is associated with the existence of an immunosuppressive network, mainly composed of MDSCs and other immunosuppressive cells that interferes with T cell trafficking and activation (Fridman et al., 2012). We therefore tested the ability of MDSCs to restrain T cell proliferation, in vitro. Prominently, we can conclude from our in vitro T cell experiments that tumor derived MDSC carried out the suppression of T cell proliferation and interferon gamma production. We report that tumor MDSCs were activated cells and were able to suppress autologous T cells and IFN-γ production in vitro as supported by previous work in colon cancer patients (Diaz-Montero et al., 2009). These findings, together with the increased expression of MDSCs in the peripheral blood, confirm the phenotypic and immunosuppressive functional characteristics of MDSCs in GC patients. 

The mechanism of suppression by MDSC might depend on the arginase pathway (Ko et al., 2010) through L-arginine depletion, as they express high levels of arginase, which in turn hydrolyzes the amino acid L-arginine to ornithine and urea. So, the depletion of such amino acid from the microenvironment profoundly inhibits T-cell function (Rodriguez et al., 2004). It was supported by findings from previous study on renal cancer patients with a correlation between increased numbers of MDSCs in the peripheral blood, low L-arginine levels in plasma, and a profound T-cell dysfunction (Zea et al., 2005). 

Controlling MDSCs accumulation using drugs, antibodies or gene ablation is a powerful tool to hinder tumor progression and enhancing antitumor immunity, and may result in tumor regression (Vincent et al., 2010; Veltman et al., 2010; Roth et al., 2012). Thus, the effectiveness of cancer immunotherapies will be improved by addressing the immunosuppressive factors associated with these cells, both systemically and in the local tumor ME. TNF-α signaling for example was proved to mediate MDSC accumulation by affecting their apoptosis rather than proliferation (Zhao et al., 2012). In this comprehensive analysis of some of these factors, we have shown a statistically significant correlation of tumor TGF-β and MDSCs in patients with GC. A major focus of this study is to assess whether tumor infiltrating levels of MDSCs correlate with TGF-β. Indeed, we found that MDSCs correlated with intra-tumor levels of TGF-β. These data suggest that this cytokine promote accumulation of MDSCs in the parenchyma of the GC. Bellone and colleagues first reported an increase in TGF-β concentrations in the sera of pancreatic cancer patients and showed anti-proliferative activity on PBMCs (Bellone et al., 1999). Of relevance are recent results on TGF-β-targeted therapy showing synergism with anti-PDL1- based treatment by dampening the tumor immunosuppressive microenvironment and favoring T cell trafficking to the tumor (Mariathasan et al., 2018). 

In this study, we extend the previous findings by analyzing miRNA-494; while these data are associative, it again suggests that miRNA-494 plays a role in the accumulation of MDSCs in GC patients and plays an important role in immunomodulation of the tumor ME. We and other investigators showed that miRNA-494 was upregulated in cancer, although a role for these molecules in immune suppression in human cancer has not been demonstrated (Zhang et al., 2018). 

Compiled data have determined that miR-494 played an important role in cancer (Romano et al., 2012 ;Yamanaka et al., 2012 ; Ramachandran et al., 2013); involving various pathophysiologic processes, including cell apoptosis, survival, tumor metastasis, and angiogenesis (Liu et al., 2010; Liu et al., 2012; Romano et al., 2012). Moreover, it was reported that miR-494 induces MDSCs activation by targeting PTEN, a major negative regulator of the PI3K/Akt signaling pathway. Thus, it appears that up-regulation of miR-494 leads to a reduction of PTEN expression, thereby resulting in an increase in Akt activity and the subsequent accumulation of functional MDSCs (Chen et al., 2015).

While we did not exhaust all the possible comparisons, we can conclude from data obtained in this study that a particularly important finding of our study is that in patients with GC, there is a significant elevation of tumor miRNA-494. High miRNA494 was associated with LN involvement and cancer stage. In addition, we showed in this study that miRNA-494 was normally expressed in the surrounding paraneoplastic tissue with no significant difference than healthy individuals.

In conclusion, tumor-derived MDSCs show functional properties (the ability to abrogate T cells) and represent a particular branch within the complexity and heterogeneity of GC microenvironment cell population. MDSCs increased with cancer stage and higher MDSC percentages correlated with poor tumor features. We do not believe that MDSCs definition is an outdated concept, but it rather defines a myeloid cell subset with unique properties, as we demonstrated in this work. Our study elucidates a possible association between MDSCs and TGF-β and microRNA-494, providing promising remedial molecular targets against tumors that may present a novel target for decreasing MDSC levels in the tumor ME. In this regard, our data open a new insight in MDSCs biology that may present a potential strategy for tumor treatment, which could enhance the efficacy of immunotherapy and targeted therapy approaches, including immune checkpoint blockade, for this lethal disease.

## References

[B1] Arihara F, Mizukoshi E, Kitahara M (2013). Increase in CD14+HLA-DR -/low myeloid-derived suppressor cells in hepatocellular carcinoma patients and its impact on prognosis. Cancer Immunol Immunother.

[B2] Batlle E, Massagué J (2019). Transforming growth factor-β signaling in immunity and cancer. Immunity.

[B3] Bellone G, Turletti A, Artusio E (1999). Tumor associated transforming growth factor-beta and interleukin-10 contribute to a systemic Th2 immune phenotype in pancreatic carcinoma patients. Am J Pathol.

[B4] Bergenfelz C, Leandersson K (2020). The generation and identity of human myeloid-derived suppressor cells. Front Oncol.

[B5] Bodogai M, Moritoh K, Lee-Chang C (2015). Immunosuppressive and prometastatic functions of myeloid-derived suppressive cells rely upon education from tumor-associated B cells. Cancer Res.

[B6] Bronte V, Brandau S, Chen SH (2016). Recommendations for myeloid-derived suppressor cell nomenclature and characterization standards. Nat Commun.

[B7] Chen S, Zhang Y, Kuzel, TM, Zhang B (2015). Regulating tumor myeloid-derived suppressor cells by MicroRNAs. Cancer Cell Microenviron.

[B8] Cui TX, Kryczek I, Zhao L (2013). Myeloid-derived suppressor cells enhance stemness of cancer cells by inducing microRNA101 and suppressing the corepressor CtBP2. Immunity.

[B9] Diaz-Montero CM, Finke J, Montero AJ (2014). Myeloid-derived suppressor cells in cancer: therapeutic, predictive, and prognostic implications. Semin Oncol.

[B10] Diaz-Montero CM, Salem ML, Nishimura MI (2009). Increased circulating myeloid-derived suppressor cells correlate with clinical cancer stage, metastatic tumor burden, and doxorubicin-cyclophosphamide chemotherapy. Cancer Immunol Immunother.

[B11] El Gazzar M, McCall CE (2012). MicroRNAs regulatory networks in myeloid lineage development and differentiation: regulators of the regulators. Immunol Cell Biol.

[B12] Fan B, Zhang LH, Jia YN 2012. Presence of S100A9-positive inflammatory cells in cancer tissues correlates with an early stage cancer and a better prognosis in patients with gastric cancer. BMC Cancer.

[B13] Freeman SD, Kelm S, Barber EK, Crocker PR (1995). Characterization of CD33 as a new member of the sialoadhesin family of cellular interaction molecules. Blood.

[B14] Fridman WH, Pagès F, Sautès-Fridman C, Galon J (2012). The immune contexture in human tumours: impact on clinical outcome. Nat Rev Cancer.

[B15] De Cicco P, Ercolano G, Ianaro A (2020). The new Era of cancer immunotherapy: Targeting myeloid-derived suppressor cells to overcome immune evasion. Front Immunol.

[B16] Fuss IJ, Kanof ME, Smith PD, Zola H (2009). Isolation of whole mononuclear cells from peripheral blood and cord blood. Curr Protoc Immunol.

[B17] Gabrilovich DI, Ostrand-Rosenberg S, Bronte V (2012). Coordinated regulation of myeloid cells by tumours. Nat Rev Immunol.

[B18] He L, Hannon GJ (2004). MicroRNAs: small RNAs with a big role in gene regulation. Nat Rev Genet.

[B19] Katz LH, Likhter M, Jogunoori W (2016). TGF-beta signaling in liver and gastrointestinal cancers. Cancer Lett.

[B20] Ko JS, Rayman P, Ireland J (2010). Direct and differential suppression of myeloid-derived suppressor cell subsets by sunitinib is compartmentally constrained. Cancer Res.

[B21] Labani-Motlagh A, Ashja-Mahdavi M, Loskog A (2020). The tumor microenvironment: A Milieu Hindering and Obstructing Antitumor Immune Responses. Front Immunol.

[B22] Law AMK, Valdes-Mora F, Gallego-Ortega D (2020). Myeloid-derived suppressor cells as a therapeutic target for cancer. Cells.

[B23] Lee CR, Kwak Y, Yang T (2016). Myeloid-derived suppressor cells are controlled by regulatory T cells via TGF-beta during Murine Colitis. Cell Rep.

[B24] Lee CR, Lee W, Cho SK, Park SG (2018). Characterization of multiple cytokine combinations and TGF-β on differentiation and functions of myeloid-derived suppressor cells. Int J Mol Sci.

[B25] Lim HX, Kim TS, Poh CL (2020). Understanding the differentiation, expansion, recruitment and suppressive activities of myeloid-derived suppressor cells in cancers. Int J Mol Sci.

[B26] Liu CY, Wang YM, Wang CL (2010). Population alterations of L-arginase- and inducible nitric oxide synthase-expressed CD11b+/CD14-/CD15+/CD33+ myeloid-derived suppressor cells and CD8+ T lymphocytes in patients with advanced-stage non-small cell lung cancer. J Cancer Res Clin Oncol.

[B27] Liu L, Jiang Y, Zhang H, Greenlee AR, Han Z (2010). Overexpressed miR-494 downregulates PTEN gene expression in cells transformed by anti-benzopyrenetrans −7, 8-dihydrodiol-9, 10-epoxide. Life Sci.

[B28] Liu Y, Lai L, Chen Q (2012). MicroRNA-494 is required for the accumulation and functions of tumor-expanded myeloid-derived suppressor cells via targeting of PTEN. J Immunol.

[B29] Luo J, Chen XQ, Li P (2019). The role of TGF-β and Its receptors in gastrointestinal cancers. Transl Oncol.

[B30] Ma P, Beatty PL, McKolanis J (2019). Circulating myeloid derived suppressor cells (MDSC) that accumulate in premalignancy share phenotypic and functional characteristics with MDSC in cancer. Front Immunol.

[B31] Mariathasan S, Turley SJ, Nickles D (2018). TGF beta attenuates tumour response to PD-L1 blockade by contributing to exclusion of T cells. Nature.

[B32] Marigo I, Bosio E, Solito S (2010). Tumor-induced tolerance and immune suppression depend on the C/EBPb transcription factor. Immunity.

[B33] Melani C, Chiodoni C, Forni G, Colombo MP (2003). Myeloid cell expansion elicited by the progression of spontaneous mammary carcinomas in c-erbB-2 transgenic BALB/c mice suppresses immune reactivity. Blood.

[B34] Najjar YG, Rayman P, Jia X (2017). Myeloid-derived suppressor cell subset accumulation in renal cell carcinoma parenchyma is associated with intratumoral expression of IL1β, IL8, CXCL5, and Mip-1α. Clin Cancer Res.

[B35] Ohki S, Shibata M, Gonda K (2012). Circulating myeloid derived suppressor cells are increased and correlate to immune suppression, inflammation and hypoproteinemia in patients with cancer. Oncol Rep.

[B36] Ramachandran S, Karp PH, Osterhaus SR (2013). Post-transcriptional gegulation of CFTR expression and function by MicroRNAs. Am J Respir Cell Mol Biol.

[B37] Rodriguez PC, Ernstoff MS, Hernandez C (2009). Arginase I-producing myeloid-derived suppressor cells in renal cell carcinoma are a subpopulation of activated granulocytes. Cancer Res.

[B38] Romano G, Acunzo M, Garofalo M (2012). 8. MiR-494 is regulated by ERK1/2 and modulates TRAIL-induced apoptosis in non–small-cell lung cancer through BIM down-regulation. Proc Natl Acad Sci U S A.

[B39] Roth F, De La Fuente AC, Vella JL (2012). Aptamer mediated blockade of IL4Rα triggers apoptosis of MDSCs and limits tumor progression. Cancer Res.

[B40] Salminen A, Kaarniranta K, Kauppinen A (2019). Immunosenescence: the potential role of myeloid-derived suppressor cells (MDSC) in age-related immune deficiency. Cell Mol Life Sci.

[B41] Santibanez JF, Bjelica S (2017). Transforming growth factor-Beta1 and myeloid-derived suppressor cells interplay in cancer. Open Cancer Immunol J.

[B42] Sitarz R, Skierucha M, Mielko J (2018). Gastric cancer: epidemiology, prevention, classification, and treatment. Cancer Manag Res.

[B43] Su Y, Qiu Y, Qiu Z (2020). MicroRNA networks regulate the differentiation, expansion and suppression function of myeloid-derived suppressor cells in tumor microenvironment. J Cancer.

[B44] Toor SM, Syed Khaja AS, El Salhat H (2016). Increased levels of circulating and tumor-infiltrating granulocytic myeloid cells in colorectal cancer patients. Front Immunol.

[B45] Toor SM, Syed Khaja AS, El Salhat H (2017). Myeloid cells in circulation and tumor microenvironment of breast cancer patients. Cancer Immunol Immunother.

[B46] Veltman JD, Lambers ME, van Nimwegen M (2010). COX 2 inhibition improves immunotherapy and is associated with decreased numbers of myeloid derived suppressor cells in mesothelioma. Celecoxib influences MDSC function. BMC Cancer.

[B47] Vignali D, Kallikourdis M (2017). Improving homing in T cell therapy. Cytokine Growth Factor Rev.

[B48] Vincent J, Mignot G, Chalmin, F (2010). 5 Fluorouracil selectively kills tumor associated myeloid derived suppressor cells resulting in enhanced T cell dependent antitumor immunity. Cancer Res.

[B49] Walter S, Weinschenk T, Stenzl A (2012). Multipeptide immune response to cancer vaccine IMA901 after single-dose cyclophosphamide associates with longer patient survival. Nat Med.

[B50] Yamanaka S, Campbell NR, An F (2012). Coordinated effects of microRNA-494 induce G (2)/M arrest in human cholangiocarcinoma. Cell Cyclen.

[B51] Zea AH, Rodriguez PC, Atkins MB (2005). Arginase-producing myeloid suppressor cells in renal cell carcinoma patients: a mechanism of tumor evasion. Cancer Res.

[B52] Zhang B, Wang Z, Wu L (2013). Circulating and tumor-infiltrating myeloid-derived suppressor cells in patients with colorectal carcinoma. PLoS One.

[B53] Zhang Y, Guo L, Li Y (2018). MicroRNA-494 promotes cancer progression and targets adenomatous polyposis coli in colorectal cancer. Mol Cancer.

[B54] Zhao X, Rong L, Zhao X (2012). TNF signaling drives myeloid derived suppressor cell accumulation. J Clin Invest.

[B55] Zhou J, Donatelli SS, Gilvary DL (2016). Therapeutic targeting of myeloid-derived suppressor cells involves a novel mechanism mediated by clusterin. Sci Rep.

